# Preventing HIV Infection in Pregnant Women in Western Uganda Through a Comprehensive Antenatal Care-Based Intervention: An Implementation Study

**DOI:** 10.1007/s10508-023-02726-z

**Published:** 2023-11-09

**Authors:** Lisa S. Jahn, Agnes Kengonzi, Steven N. Kabwama, John Rubaihayo, Stefanie Theuring

**Affiliations:** 1 Institute of International Health, Charité - Universitätsmedizin Berlin, Corporate Member of Freie Universität Berlin and Humboldt-Universität Zu Berlin, Augustenburger Platz 1, 13353 Berlin, Germany; 2 School of Health Sciences, Mountains of the Moon University, Fort Portal, Uganda; 3 School of Public Health, Makerere University, Kampala, Uganda

**Keywords:** HIV prevention, Pregnancy, HIV risk behavior, Partner involvement, Repeat testing

## Abstract

We implemented and assessed a comprehensive, antenatal care (ANC)-embedded strategy to prevent HIV seroconversions during pregnancy in Uganda. HIV-negative first-time ANC clients were administered an HIV risk assessment tool and received individual risk counseling. Those attending ANC without partners obtained formal partner invitation letters. After three months, repeat HIV testing was carried out; non-attending women were reminded via phone. We analyzed uptake and acceptance, HIV incidence rate, and risk behavior engagement. Among 1081 participants, 116 (10.7%) reported risk behavior engagement at first visit; 148 (13.7%) were accompanied by partners. At the repeat visit (*n* = 848), 42 (5%, *p* < 0.001) reported risk behavior engagement; 248 (29.4%, *p* < 0.001) women came with partners. Seroconversion occurred in two women. Increased odds for risk behavior engagement were found in rural clients (aOR 3.96; 95% CI 1.53–10.26), women with positive or unknown partner HIV-status (2.86; 1.18–6.91), and women whose partners abused alcohol (2.68; 1.15–6.26). Overall, the assessed HIV prevention strategy for pregnant women seemed highly feasible and effective. Risk behavior during pregnancy was reduced by half and partner participation rates in ANC doubled. The observed HIV incidence rate was almost four times lower compared to a pre-intervention cohort in the same study setting.

## Introduction

According to the UNAIDS Update 2021, the HIV pandemic is far from over, although remarkable progress has been made (UNAIDS, 2021; Vos et al., 2020; WHO, 2021). The incidence in the general population has largely stabilized, but key populations are still at high risk of HIV infection (UNAIDS, 2021; WHO, 2016, 2017, 2021). Two-thirds of all people with HIV are living in sub-Saharan Africa (UNAIDS, 2021) and 10% in Uganda (WHO, 2016), where the HIV prevalence rate reaches 5.4% (Brahmbhatt et al., 2019; UNAIDS, 2018). Especially in countries with high prevalence rates like Eswatini, Lesotho or Botswana, but also Uganda, large numbers of pregnant and lactating women become infected with HIV, and mother-to-child transmission (MTCT) is responsible for over 10% of global HIV infections (Moodley et al., 2009; Statista, 2023; WHO, 2016, 2017, 2021)**.** Certain biological factors can raise pregnant women´s vulnerability to infection: Increased progesterone levels are associated with cervical inflammation, which is favored by altered vaginal flora and leads to weakening of the immune system (WHO, 2017). Additionally, social and behavioral changes during pregnancy, like omitted condom use, may increase the risk of exposure (Musekiwa et al., 2013; WHO, 2017, 2021)**.**

The World Health Organization (WHO) describes essential strategies to avoid HIV infection, including condom use, oral pre-exposure prophylaxis (PrEP) and post-exposure prophylaxis. Scaling-up HIV testing could greatly contribute to ending the pandemic, because targeted prevention and early therapy can be initiated (FHI360, 2017; WHO, 2016, 2017, 2021). Such strategies should also be promoted in antenatal care (ANC) to prevent HIV infection in pregnant women. However, they are usually not routinely offered, and no specific guidelines exist for protecting women who have tested HIV-negative at the beginning of pregnancy (Mbuagbaw et al., 2015; Tudor Car et al., 2011; UNAIDS, 2021; WHO, 2017, 2020, 2021).

It is assumed that pregnant women are at particular risk to be newly infected. In 2017, a study from Western Uganda showed that the HIV incidence rate among pregnant women was three times higher compared with the general female population (Schumann et al., 2020). With 4.8. children per woman, Uganda has one of the highest fertility rates worldwide (UNDATA, 2021) and more than 90% of pediatric new infections occur through MTCT (MoH Uganda, 2018)**.**

The Ministry of Health in Uganda follows a "test and treat" strategy to reduce new HIV infections (MoH Uganda, 2018, 2020) and pregnant women are offered HIV testing as part of ANC, but there are no specifically targeted measures to prevent their seroconversion.

The aim of this study was to evaluate an evidence-based prevention strategy for pregnant women to prevent new HIV infections in Western Uganda.

## Method

For this implementation research, we conducted a longitudinal cohort study among pregnant women in Fort Portal, Kabarole District between June 2020 and February 2021.

In HIV prevention, it is recognized that a combination of complementary evidence-based strategies at the behavioral, biomedical, and structural levels is most effective (UNAIDS, 2007), and the Ministry of Health Uganda (2018) has proclaimed HIV combination prevention as a target strategy. For this reason, the prevention approach in this study consisted of several highly specific interventions tailored to the individual needs of women.

The basis for developing our intervention was a preceding study (Schumann et al., 2020) in the same three health facilities, where several risk factors for HIV seroconversion during pregnancy were identified. Our risk assessment tool was informed by those factors.

The 2020 study indicated that among women who were tested HIV-negative at the first ANC examination, only a brief counseling about general HIV prevention was offered afterwards. However, other research suggests that general HIV counseling has little impact on HIV-preventive behavior (Johnson et al., 2012). Counseling must address the specific situation and individual risk of the person in order to effect potential behavior change, which we incorporated in the implemented counseling strategy. As another aspect for developing our intervention, only few women in the baseline study were accompanied by their partners to ANC. Consideration of partner serostatus and joint couple counseling in case of serodiscordance is a particularly important risk reduction strategy for HIV-negative pregnant women (MoH Lesotho, 2014), while it has been shown that male partner involvement in ANC is complex. In a previous study in Tanzania, formal invitation letters increased male partner return from 2 to 31% (Theuring et al., 2016); we therefore included this approach in our intervention.

Fort Portal is a city located in Kabarole District, Western Uganda, and is home to approximately 53.000 people (Uganda Bureau of Statistics, 2017). Kabarole District has a population of 318.000 and an HIV positive rate of 3.2%, while the incidence per 1.000 among adults (15–49 years) in whole Uganda is 1.72. Women account for more than 58% of HIV cases (UNAIDS, 2021; UNDATA, 2021).

For our multicenter study, three health facilities were purposively selected to cover diverse socioeconomic segments of society in a maximum variation approach: the rural public Kibiito Health Center IV (about 30 km outside Fort Portal city), the public urban Fort Portal Regional Referral Hospital in Buhinga, and the private catholic Holy Family Virika Hospital within Fort Portal city. All three facilities offer free ANC and maternity care, as well as prevention of MTCT (PMTCT) and HIV treatment services.

### Participants

As a standard procedure, first-time ANC clients are routinely tested for HIV, and, if positive, enrolled in PMTCT care. Women who are HIV-negative are advised to be tested again after three months, but this is not routinely enforced (Larsson et al., 2012). No other HIV prevention measure is applied for HIV-negative pregnant women.

Women who had a confirmed pregnancy, had not exceeded 28 gestational weeks, and were tested HIV-negative at first ANC encounter were eligible for recruitment if they were at least 15 years old (UNCST, 2014) and provided written informed consent.

### Measures and Procedure

After routine ANC, trained local study nurses conducted structured interviews using a self-developed and pilot-tested questionnaire, covering sociodemographic, health- and behavior-related information of the women. Additionally, participants were asked to self-assess their perceived risk for HIV infection on a four-point scale (“high” to “not at all”). Subsequently to filling in the questionnaire, study staff carried out the comprehensive, three-step HIV prevention intervention. The first step included application of a systematic individual HIV risk assessment tool. According to their individual risk, women received solution-oriented counseling. Specifically, the trained counselor discussed with the participant how pregnancy increases vulnerability to HIV infection, the personal risk concerns and risk reduction strategies. The solution-oriented approach aimed at directly offering feasible options to the client for reducing individual risk, including condom negotiation skills or immediate referral to PrEP services if PrEP eligibility criteria according to National Guidelines (MoH Uganda, 2018) applied. The counselors were trained to use insightful listening skills and supportive discussion to enable the participants to open up, share their concerns and agree on strategies to reduce the risk of infection. As a second step of the intervention, women who were not accompanied by their partner at first ANC visit received an official invitation letter for their partner for a couples´ counseling and testing session. As a third step, women were given an appointment for the repeat HIV test after three months, aligned with their routine ANC visit schedule.

This repeat test date served as follow-up time point for the study. After routine ANC, study nurses filled in a follow-up questionnaire for the women to retrieve HIV-related risk behavior during pregnancy in the time having elapsed since recruitment. Women were routinely re-tested for HIV, and the result was noted. We also captured if partner invitation letters had been handed out and if partners had complied with them. Women who did not appear for this appointment were reminded via text message or phone call. If they showed up for their new appointment, this served as the follow-up time point for assessing the impact of the intervention.

The socioeconomic status (SES) was measured by a score from zero to nine as used in other thematically related publications (Schnack et al., 2016; Schumann et al., 2020; Theuring et al., 2021) depending on nine common potential properties, uniformly dichotomized and categorized in “lower” scoring zero to four, or “higher” scoring five to nine.

We created a dichotomous variable for risk behavior engagement including sex with HIV-positive person, unprotected sex with person of unknown HIV status, alcohol abuse, sex under influence of alcohol, sex with someone under influence of alcohol or drugs, trading sex for goods or benefits, intravenous drug use and commercial sex work. Women who reported at least one of those behaviors during the past year were categorized as “engaging in risk behavior.” Partner serostatus was included in the questionnaire as an independent sociodemographic variable but was not part of the definition of risk behavior engagement, because the official partner of a woman is not always congruent with her sex partner(s).

### Data Analysis

The dataset was anonymized and entered into Excel. The data were cleaned, and duplicate entries were eliminated. The incidence rate was determined by seroconversions in pregnant women per 100 person-years. Statistical analysis was performed using SPSS. For descriptive analysis, absolute and relative numbers were reported. Metric data were represented by mean, range, and standard deviations. To assess differences between women who did or did not engage in risky behavior before and after the intervention, univariate data analysis was performed. For univariable logistic regression, odds ratios (OR) and 95% confidence intervals (95% CI) were reported. For multivariable logistic regression, a model with variables of interest was created. Variables were removed using backwards elimination up to an Akaike information criterion of 0.157 to estimate how well the model represents the data (Heinze et al., 2018). For this, adjusted odds ratios (aOR) and 95% CIs were reported.

## Results

### Sociodemographic Background of the Study Population

In total, 1081 females participated in our study with a mean age of 25.3 years. We recruited 577 (53.4%) women in the urban public Buhinga Hospital (mean SES, 5), 323 (29.9%) in the rural public Kibiito Health Center (mean SES, 4), and 181 (16.7%) in the urban private Virika Hospital (mean SES, 6). Personal risk of HIV infection was assessed as “not present” by 392 (36.3%) women and as “low” by 350 (32.4%). Only 68 (6.3%) considered themselves to be at “high risk.” The partner´s HIV status was unknown to 117 (10.8%) women, 15 (1.4%) knew their partner was HIV-positive.

Having a partner with more than one sexual relationship or being unsure about it was reported by 342 (31.7%) women, 149 (13.8%) partners abused alcohol, eight (0.7%) visited sex workers, and four (0.4%) took intravenous drugs (Table 1).

**Table 1:** Participants´ sociodemographic and behavioral characteristics, and associations with pre-intervention engagement in risky behavior

Variable	Total (*n* = 1081) *n* (%)	Pre-intervention risk engagement (*n* = 116) n (%)	OR^a^
			(95% CI)^b^
Age	1081	116	0.99 (0.93; 1.05)
*mean, sd (range)*	25.3, 5.9 (15–45)	24.8, 6.6 (16–43)^c^	
Marital status	1081	116	
Married or couple	924 (85.5)	86 (9.3)	Reference
Single or divorced	157 (14.5)	30 (19.1)	**2.3 (1; 3.63)**
Completed education	1079	116	
None	42 (3.9)	4 (9.5)	Reference
Primary	512 (47.4)	74 (14.5)	1.61 (0.56; 4.63)
Secondary	393 (36.4)	36 (9.2)	0.96 (0.32; 2.84)
Tertiary	132 (12.2)	2 (1.5)	**0.15 (0.03; 0.83)**
Employment	1079	116	
Formal	198 (18.4)	13 (6.7)	Reference
Informal	881 (81.6)	103 (11.7)	**1.88 (1.04; 3.43)**
Wealth Score	1081	116	
Lower SES	461 (42.6)	57 (12.4)	1.34 (0.91; 1.97)
Higher SES	620 (57.4)	59 (9.5)	Reference
Facility	1081	116	
Buhinga	577 (53.4)	75 (13)	1.16 (0.76; 1.76)
Virika	181 (16.7)	4 (2.2)	**0.18 (0.06; 0.5)**
Kibiito	323 (29.9)	37 (11.5)	Reference
Gestational week	1050	114	1.01 (0.97; 1.05)
*mean, sd (range)*	18.6, 5.33 (4–30)	18.7, 4.98 (8–28)^d^	
Gravida	1081	116	**1.27 (1.05; 1.54)**
*mean, sd (range)*	2.7, 1.78 (1–12)	2.7, 1.66 (1–8)^e^	
No. of children < 15 living in household	1077	116	1.01 (0.87; 1.18)
*mean, sd (range)*	1.6, 1.55 (0–7)	1.6, 1.53 (0–7)^f^	
Partner age	1079	116	0.99 (0.95; 1.04)
*mean, sd (range)*	30.9, 7.43 (17–62)	30.2, 8.12 (17–62)^g^	
Partnership duration (years)	1073	114	**0.9 (0.85; 0.96)**
*mean, sd (range)*	5.2, 5.21 (0–28)	4.3, 5.27 (0–22)^h^	
Couple age difference	1080	111	1 (0.96; 1.05)
*mean, sd (range)*	5.9, 4.56 (0–32)	6.0, 5.49 (0–32)^i^	
HIV risk perception	1080	116	
High	68 (6.3)	16 (23.5)	**3.35 (1.72; 6.5)**
Some	279 (25)	38 (13.6)	**1.78 (1.09; 2.92)**
Very low	350 (32.4)	29 (8.3)	0.98 (0.58; 1.66)
No risk	392 (36.3)	33 (8.4)	Reference
Partner HIV status	1081	116	
Known negative	949 (87.8)	47 (5)	Reference
Unknown	117 (10.8)	59 (50.4)	**19.52 (12.25; 31.12)**
Known positive	15 (1.4)	10 (66.7)	**38.38 (12.61; 116.8)**
Sexually active	1072	115	
Yes	880 (81.4)	98 (11.1)	Reference
No	192 (17.8)	17 (8.9)	0.78 (0.45; 1.33)
No. of sex partners past year	1080	116	**5.03 (2.93; 8.64)**
*mean, sd (range)*	1.1, 0.69 (1–20)	1.5, 1.98 (1–20)^j^	
Condom use	880	98	
Always	6 (0.6)	2 (33.3)	4.62 (0.83; 25.64)
Sometimes	37 (3.4)	11 (29.7)	**3.91 (1.86; 8.22)**
Rarely	36 (3.3)	5 (13.9)	1.49 (0.56; 3.95)
Never	801 (74.1)	80 (10)	Reference
Any STD past year	1080	116	
Yes	89 (8.2)	11 (12.4)	1.19 (0.61; 2.31)
No	991 (91.7)	105 (10.6)	Reference
Partner repression/violence^**k**^	1080	115	
Yes	122 (11.3)	31 (25.4)	**3.54 (2.23; 5.64)**
No	958 (88.7)	84 (8.8)	Reference
Partner > 1 sexual relation	1081	116	
Yes/ Unknown	342 (31.6)	58 (17.0)	0.63 (0.3; 1.32)
No	739 (68.4)	58 (7.9)	Reference
Partner alcohol abuse	1080	115	
Yes	149 (13.8)	54 (36.2)	**3.71 (1.87; 7.36)**
No	931 (86.2)	61 (6.6)	Reference
Partner intravenous drug use	1079	115	
Yes	4 (0.7)	1 (25.0)	–
No	1075 (99.3)	114 (10.6)	–
Partner visits sex worker	1080	115	
Yes	8 (0.7)	3 (37.5)	–
No	1072 (99.3)	112 (10.5)	–

Numbers in bold indicate non-overlapping CI levels

^a^OR = Odds ratio; from univariate logistic regression

^b^95% CI = 95% Confidence Interval

^c^Comparison values for group without pre-intervention risk behavior: mean 25.3, sd 5.8 (range 15–45)

^d^Comparison values: 18.5, 5.4 (4–30)

^e^Comparison values: 2.7, 1.8 (1–12)

^f^comparison values: 1.6, 1.5 (0–7)

^g^Comparison values: 30.9, 7.3 (17–60)

^h^Comparison values: 5.3, 5.2 (0–28)

^i^Comparison values: 5.9, 4.4 (0–31)

^j^Comparison values: 1, 0.2 (1–3)

^k^Physical violence, emotional or financial repression

### Pre-Intervention Risky Behavior

**Fig. 1:**
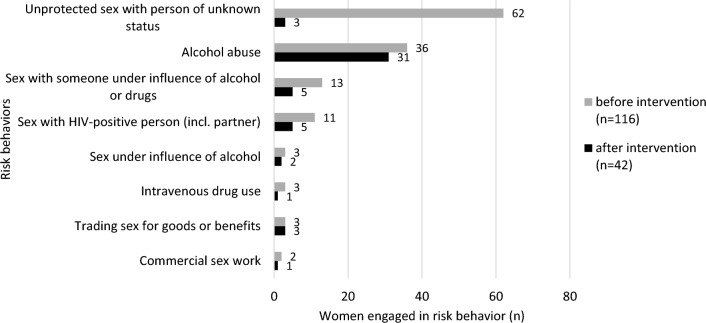
Specific risky behaviors among HIV-negative pregnant women before (*n* = 116/1081) and after (*n* = 42/844) the intervention

At recruitment, 965/1081 (89.3%) participants did not engage in risk behavior, while 116 (10.7%) did (two missing data; for detailed risk behaviors see Fig. 1). Regarding sociodemographic associations with engagement in risk behavior (Table 1), single or divorced status was a significant factor (OR 2.3; 95% CI 1.0; 3.63). Women with risk behavior engagement had less likely completed tertiary school (OR 0.15, 95% CI 0.03; 0.83) compared to no completed education, were more likely to have an informal employment (OR 1.88, 95% CI 1.04; 3.43) and less likely to be recruited in the urban private Virika Hospital (OR 0.175, 95% CI 0.06; 0.498) compared to the public rural Kibiito Health Center. Odds of engagement in risk behavior decreased with each year of relationship duration (OR 0.90, 95% CI 0.85; 0.96). Higher number of pregnancies in a woman was associated with increased odds for risky behavior (OR 1.27, 95% CI 1.05; 1.54). Women engaging in risk behavior were more likely to rate their personal HIV risk as high (OR 3.35, 95% CI 1.72; 6.50) and to report partner HIV status as unknown (OR 19.52, 95% CI 12.25; 31.12) or positive (OR 38.38, 95% CI 12.61; 116.8). Partner´s alcohol abuse was significantly more common among women engaging in risk behavior (OR 3.71, 95% CI 1.87; 7.36).

### Uptake and Acceptability of the Preventive Intervention

As a result of the individual risk counseling, 34/1081 (3.2%) women qualified for PrEP at recruitment, of which 33 were referred directly to PrEP services. Eight of these were lost to follow-up. Three of 33 (9.1%) presented at PrEP services, where it was initiated for two (6.1%). The third woman was already taking PrEP drugs regularly. Of the newly initiated women, one did not take the drugs because she self-reported forgetting to do so, and one took the drugs most of the time.

The first ANC appointment was attended by 148/1081 partners (13.7%), of whom 139 (12.9%) were tested for HIV that day; 931 (86.1%) women came unaccompanied (two missing data). Invitation letters for partners were given to 899/931 (96.6%) women.

In total, 848/1081 (78.5%) women participated in the follow-up visit after three months, of whom four (0.4%) had to be excluded because of missing data; 233 (21.6%) did not return for repeat testing. From the 844 returning women, 204 (24.2%) had initially missed their repeat test appointment and had been successfully reminded. Among the 844 returning women, 765 (90.6%, two missing data) had received a partner invitation letter at the first ANC visit, of whom 720 (94.1%) reported they had delivered it to their partners. A positive attitude toward the letters was reported for 638/765 (83.4%) partners. At follow-up, 248/844 (29.4%) partners visited ANC, a significant increase to baseline (Wilcoxon rank-sum test *p* < 0.001); of these, 245 (98.8%) were tested for HIV. After partner testing, 4/245 women (1.6%) reported their partner had tested positive, and 66 (26.9%) did not know the test result, while 175 (71.4%) had received a negative partner test result.

Seroconversion between the two study visits was detected in two women (0.2%) out of 844. This corresponds to an incidence rate of 0.76/100 person-years.

### Post-Intervention Risk Behavior

In the follow-up cohort, 42/844 women (5.0%) reported engagement in risk behavior (details see Fig. 1), a significant decrease compared to the baseline value (10.7%; Wilcoxon rank-sum test *p* < 0.001, two missing data). Sociodemographic and behavioral associations with post-intervention engagement in risk behavior included a lower SES (OR 3.27, 95% CI 1.68; 6.4), unknown partner HIV status (OR 3.56; 95% CI1.66; 7.64) or known HIV-positive partner (OR 22.25, 95% CI 6.39; 77.47), as well as partner´s alcohol abuse (OR 3.32, 95% CI 1.69; 6.51). Tertiary level education was linked with lower odds of risk behavior engagement (OR 0.1, 95% CI 0.01; 0.99) (Table 2).

**Table 2:** Follow-up cohort: sociodemographic and behavioral characteristics and associations with post-intervention engagement in risky behavior

Variable	Total (*n* = 844)	Post-intervention risk engagement (*n* = 42)	OR^a^
	*n* (%)	*n* (%)	(95% CI)^b^
Age	844	42	1 (0.95; 1.06)
*mean, sd (range)*	25.4, 6.13 (15–45)	25.4, 6.52 (17–42)^c^	
Marital status	844	42	
Married or couple	742 (87.9)	34 (4.6)	Reference
Single or divorced	102 (12.1)	8 (7.8)	0.56 (0.25; 1.26)
Completed education	842	42	
None	35 (4.2)	3 (8.6)	Reference
Primary	388 (46.1)	28 (7.2)	–
Secondary	311 (36.9)	10 (3.2)	–
Tertiary	108 (12.8)	1 (0.9)	**0.1 (0.01; 0.99)**
Employment	844	42	
Formal	154 (18.3)	2 (1.3)	Reference
Informal	690 (81.8)	40 (5.8)	**1.54 (1.12; 19.56)**
Wealth Score	844	42	
Lower SES	354 (41.9)	29 (8.2)	**3.27 (1.68; 6.4)**
Higher SES	490 (58.1)	13 (2.7)	Reference
Facility	844	42	
Buhinga	372 (44.1)	9 (2.4)	**0.21 (0.1; 0.45)**
Virika	168 (19.9)	1 (0.6)	**0.05 (0.01; 0.38)**
Kibiito	304 (36)	32 (10.5)	Reference
Gestational week	820	42	
*mean, sd (range)*	18.3, 4.94 (4–30)	18.4, 4.54 (6–28)^d^	1.01 (0.95; 1.07)
Gravida	844	42	1.09 (0.92; 1.28)
*mean, sd (range)*	2.8, 1.7 (1–12)	3, 1.7 (1–12)^e^	
No. of children < 15 living in household	841	42	1.08 (0.89; 1.31)
*mean, sd (range)*	1.7, 1.49 (0–7)	1.8, 1.4 (0–6)^f^	
Partner age	843	42	1.01 (0.96; 1.05)
*mean, sd (range)*	31.3, 7.59 (17–62)	31.5, 7.87 (21–50)^g^	
Partnership duration (years)	838	42	1.02 (0.96; 1.07)
*mean, sd (range)*	5.6, 6.1 (0–28)	5.8, 6.94 (0–28)^h^	
Couple age difference	843	42	1.04 (0.97; 1.1)
*mean, sd (range)*	6, 5.26 (0–32)	6.8, 4.65 (0–22)^i^	
Risk perception of getting HIV	843	42	
High	45 (5.3)	7 (15.6)	**5.06 (1.85; 13.83)**
Some	222 (26.3)	15 (6.8)	1.99 (0.9; 4.42)
Very low	263 (31.2)	9 (3.4)	0.97 (0.4; 2.39)
No risk	313 (37.1)	11 (3.5)	Reference
Partner HIV status	844	42	
Known negative	748 (88.6)	27 (3.6)	Reference
Unknown	85 (10.1)	10 (11.8)	**3.56 (1.66; 7.64)**
Known positive	11 (1.3)	5 (45.5)	**22.25 (6.39; 77.47)**
Sexually active	836	42	
Yes	691 (82.7)	34 (4.9)	Reference
No	145 (17.3)	8 (5.5)	1.13 (0.51; 2.29)
No. of sex partners past year	844	42	0.8 (0.25; 2.61)
*mean, sd (range)*	1.1, 0.32 (1–10)	1.1, 0.22 (1–2)^j^	
Condom use	696	35	
Always	7 (1)	1 (14.3)	3.6 (0.42; 30.94)
Sometimes	28 (4)	4 (14.3)	**3.6 (1.17; 11.08)**
Rarely	28 (4)	2 (14.3)	1.66 (0.38; 7.36)
Never	633 (91)	28 (4.4)	Reference
Any STD past year	843	42	
Yes	71 (8.4)	3 (4.2)	0.83 (0.25; 2.75)
No	772 (91.6)	39 (5.1)	Reference
Partner repression /violence^k^	843	42	
Yes	78 (9.3)	3 (3.9)	1.34 (0.41; 4.45)
No	765 (90.8)	39 (5.1)	Reference
Partner > 1 sexual relationship	844	42	
Yes/Unknown	250 (29.6)	10 (4)	0.73 (0.35; 1.51)
No	594 (70.4)	32 (5.4)	Reference
Partner alcohol abuse	844	42	
Yes	119 (14.1)	14 (11.8)	**3.32 (1.69; 6.51)**
No	725 (85.9)	28 (3.9)	Reference
Partner intravenous drug use	843	42	
Yes	2 (0.2)	0 (0)	
No	841 (99.8)	42 (5)	–
Partner visits sex worker	844	42	
Yes	5 (0.6)	0 (0)	
No	839 (99.4)	42 (5)	–

Numbers in bold indicate non-overlapping CI levels

^a^OR = Odds Ratio; from univariate logistic regression

^b^95% CI = 95% Confidence Interval

^c^Comparison values, group without post-intervention risk behavior: mean 25.4, sd 5.7 (range 15–45)

^d^Comparison values: 18.2, 5.4 (4–30)

^e^Comparison values: 2.7, 1.8 (1–10)

^f^Comparison values: 1.6, 1.6 (0–7)

^g^Comparison values: 31.2, 7.3 (17–62)

^h^Comparison values: 5.4, 5.2 (0–28)

^i^Comparison values: 6, 4.5 (0–32)

^j^Comparison values: 1.1, 0.4 (1–10)

^k^Physical violence, emotional or financial repression

Partner attendance at the first ANC visit was not significantly associated with engagement in risk behavior during pregnancy (p = 0.69). However, among women not engaging in risk behavior at follow-up (*n* = 802), partner attendance had increased after the intervention (recruitment: 116/802, 14.5% vs. follow-up: 241/802, 30.0%), while in those women engaging in risk behavior at follow-up (*n* = 42), partner attendance had decreased (recruitment: 7/42, 16.7% vs. follow-up: 4/42, 9.5%). Women not engaging in risk behavior at recruitment had handed out the invitation letter more often (p = 0.02), and their partners attended ANC more often at follow-up (p = 0.004). Partner’s reaction to the letter was significantly less supportive by partners of woman engaging in risk behavior (p = 0.017).

In multivariable logistic regression analysis, being a client at Kibiito Health Center (AOR 3.96, 95% CI 1.53, 10.26) compared to Buhinga Hospital, having a partner with positive or unknown HIV status (AOR 2.86, 95% CI 1.18, 6.91) compared to an HIV-negative partner, and partner alcohol abuse (AOR 2.68, 95% CI 1.15, 6.26) were independently associated with risk behavior engagement among pregnant women who had received the preventive intervention (Table 3).

**Table 3:** Factors associated with engagement in risky behavior in follow-up by multivariate logistic regression

Variable	Post-intervention risk engagement (*n* = 42) n (%)	AOR^a^ (95% CI)^b^
Facility		
Kibiito (*n* = 272)	32 (11.8)	**3.96 (1.53, 10.26)**
Buhinga (*n* = 363)	9 (2.5)	Reference
Virika (*n* = 167)	1 (0.6)	–
Number of people living in household *mean, sd (range)*	4.2, 1.79 (1–9)^c^	2.18 (0.72; 6.59)
Partner HIV status		
Positive, Unknown (*n* = 96)	15 (15.6)	**2.86 (1.18; 6.91)**
Negative (*n* = 748)	27 (3.6)	Reference
Partner alcohol abuse		
Yes (*n* = 119)	14 (11.8)	**2.68 (1.15; 6.26)**
No (*n* = 697)	28 (4)	Reference
Physical or emotional partner repression or violence		
Any repression (*n* = 78)	3 (3.9)	0.24 (0.04; 1.46)
No repression (*n* = 765)	39 (5.1)	Reference

Numbers in bold indicate non-overlapping CI levels

^a^AOR = adjusted odds ratio

^b^CI = confidence interval

^c^Comparison values, group without post-intervention risk behavior: mean 4.1, sd 2.25 (range 1–20)

## Discussion

Our study assessed a systematically implemented HIV prevention intervention for HIV-negative ANC clients in sub-Saharan Africa. We used a risk assessment tool to identify pregnant women at risk for HIV seroconversion, representing a widely used and proven method for systematic risk screening (Pintye et al., 2017; Schumann et al., 2020; UNAIDS, 2021). The self-assessed individual risk of pregnant women largely matched the HIV risk determined by our tool, showing that women had a realistic perception of their behavior. This confirmed previous studies from Uganda, where women were aware of the risks their behavior evoked (González et al., 2019; Theuring et al., 2021). Practicing risky behaviors despite being fully aware of it may indicate a general lack of knowledge regarding the consequences of maternal HIV infection for the unborn child, pointing to a need for specific education in this context (Homsy et al., 2019; Schumann et al., 2020; UNAIDS, 2021; WHO, 2017).

The components of the prevention intervention showed high acceptability and effectiveness among our study population. Compared to recruitment, the proportion of women reporting engagement in risk behavior three months after the intervention had halved. We found an overall HIV incidence rate of 0.76/100 person-years in pregnant women having received the preventive intervention. In comparison, a pre-intervention pregnant cohort in the same three health facilities in 2017 displayed an almost fourfold HIV incidence rate of 2.85/100 person-years (Schumann et al., 2020). Although comparisons between groups across different time points require some caution with respect to changes of external conditions, general HIV prevention policies in ANC had not changed in our setting between 2017 and 2020. Hence, our results suggested that the implemented preventive intervention had a positive effect on HIV prevention during pregnancy. To verify effectiveness of the prevention measure in terms of reduced HIV incidence rates, application on a more extensive scale is required.

Only one in ten PrEP-referred women presented at PrEP services. Correspondingly, uptake of this preventive measure was found to be low in other African settings (Drake et al., 2014; Kinuthia et al., 2015). It has been shown before that pregnant women can be hesitant to expose their unborn child to drugs not perceived as essentially needed (Bailey et al., 2018; Ceulemans et al., 2019). More intensive support programs and education regarding the fact that pregnancy outcomes with PrEP use are not negatively affected could increase acceptance (Townsend et al., 2008; White et al., 2014; WHO, 2017). To improve uptake, PrEP service offer could be integrated directly into ANC, following the successful model of PMTCT-ANC service integration (Turan et al., 2015).

Only one in seven women were accompanied to the first ANC visit by their partner. Acceptability of partner invitation letters was high, with most women delivering them to their partners and reporting favorable partner reaction. Yet again, only a third of the addressed partners attended ANC at the partner counseling appointment. A qualitative study from Uganda investigating parent role distribution and motivation for childbirth showed that pregnancy and childcare are still viewed as women´s tasks (Beyeza-Kashesy et al., 2010), and this traditional view of female responsibility for children could explain the low acceptance of partner testing (Chanyalew et al., 2021; Jeremiah et al., 2021; Lavender et al., 2019). However, it is important to acknowledge that after the intervention, the partner return rate had doubled as compared to baseline.

Because biological and behavioral changes during pregnancy can increase the risk of HIV exposure, it is important to repeat HIV testing as part of ANC, as also recommended in the Ugandan National guidelines (MoH Uganda, 2018). However, in a setting like our study, repeat HIV testing is often not routinely performed due to overburdened staff in ANC and delivery wards (Kassaw et al., 2020; Raru et al., 2022). Before our intervention, pregnant women had not been specifically addressed regarding repeat HIV tests, and it is encouraging that a quarter of the women presenting for repeat testing had been motivated by the simple approach of reminder text messaging or phone call, while they would otherwise have been lost to care.

Women being informally employed and having a lower SES were more likely to engage in HIV risk behavior during pregnancy. Our results correspond to various studies confirming a higher risk of HIV infection in the low-income population (Mendenhall et al., 2017; Oni et al., 2014; Schumann et al., 2020) and associations between lower income and higher HIV risk (Moodley et al., 2009; Torres et al., 2021; Wojcicki, 2005). It is important to purposively target these women and educate them on risks and their individual options for action.

In multivariable logistic regression, we found clients of rural public Kibiito Health Center to have a 4-times higher chance of post-intervention risk behavior engagement compared to the urban public Buhinga Hospital. Other studies showed a higher HIV prevalence in urban areas (Cane et al., 2021; Hargreaves et al., 2013), but young people in rural areas were more likely to report having sex under the age of 18 and rates of condom use were lower than in urban areas (Cane et al., 2021). In rural areas, the infrastructure is usually poorer with more access barriers to the health system, which may have led to increased risk behavior among Kibiito clientele. It is also possible that the connection between lower SES, as found in Kibiito, and rural life plays a role.

Intimate partner violence has previously been described as a risk factor for HIV infection (Meskele et al., 2021). One in nine women of our cohort experienced domestic repression or violence, but we found no significant association with engagement in HIV risk behavior. The Ugandan Bureau of Statistics (2018) stated that about 51% of Ugandan women have experienced physical violence. Partner repression and violence could have been underreported in our study, because affected women might avoid public health services or might be uncomfortable admitting that they suffer from domestic violence.

There were several limitations in our study. Firstly, we did not employ a simultaneous control group. It was not justifiable to offer a comprehensive HIV prevention intervention to only a part of the recruited pregnant women, while we had a time-staggered control group at disposal from our 2017 baseline study, where we assessed HIV incidence in pregnancy in the absence of any intervention in the same three facilities. However, general policies and conditions in the region had not changed over the course of the two study time points, and basic characteristics are comparable between the two cohorts (Schumann et al., 2020); we therefore assume that the comparison was justified. Secondly, women might not have answered truthfully on sensitive topics because of fear of being stigmatized. This may have resulted in underreporting of risky behavior and social desirability bias. To minimize this limitation, study personnel received special training, and emphasis was placed on a familiar and undisturbed setting. Thirdly, since seroconversion occurred in only two women, we could not analyze risk factors for actual seroconversion despite the intervention, which would require a larger cohort. Also, we experienced loss to follow-up in 21.6% of the originally recruited women, which is a frequent challenge in field studies, even more so in resource-limited settings. However, our sample size was still sufficiently large to draw conclusions regarding the feasibility and effectiveness of the intervention.

### Conclusions

We conducted one of the first implementation studies to evaluate a comprehensive intervention to prevent new HIV infections among pregnant women in Uganda. After the intervention, we observed an HIV incidence rate almost four times lower compared to a previous non-intervention cohort in the same study setting. Engagement in risk behavior during pregnancy had halved at follow-up three months after the intervention. Our research showed that a combined prevention intervention, comprising individual HIV risk assessment and counseling including PrEP referral, partner involvement in ANC and enforced repeat HIV testing, might be a highly acceptable and effective strategy to reduce HIV seroconversions in pregnancy. Pregnant women in rural settings, as well as women experiencing precarious partner situations like unknown or positive partner serostatus or partner alcohol abuse, should receive special attention with respect to HIV prevention in pregnancy. As pregnant women generally are a high at-risk group for HIV seroconversion, current efforts in sub-Saharan Africa to prevent HIV infection could benefit from introducing this highly feasible prevention strategy.

## Data Availability

The dataset was password-protected and only accessible to authorized study staff.
